# Effects of Contamination with Selected Polymers on the Mechanical Properties of Post-Industrial Recycled Polypropylene

**DOI:** 10.3390/polym16162301

**Published:** 2024-08-14

**Authors:** Michael Dawoud, Iman Taha

**Affiliations:** Sustainable Materials in Polymer Engineering, Aalen University, Beethovenstraße 1, 73430 Aalen, Germany; michael.dawoud@hs-aalen.de

**Keywords:** recycling, polypropylene, mis-sorting, mechanical behaviour, melt volume rate

## Abstract

The effect of contamination of polypropylene (PP) with selected polymers is studied to simulate the effect of mis-sorting in recycling streams. Polystyrene (PS), polyethylene terephthalate (PET), polycarbonate (PC), acrylonitrile butadiene styrene (ABS), and polylactic acid (PLA) were compounded with PP at different concentrations varying between 3 and 10%. Infrared spectroscopy proved the absence of chemical bonds between the constituents. Generally, melt flowability, except for the PP/PLA blend, and crystallinity were only slightly affected by the incorporation of the contaminating polymers. Samples of the polymer blends were injection moulded and further tested for their tensile and impact properties. Critical behaviour was induced by the introduction of a weld line as a result of the application of multiple gating points during injection moulding. Results generally show the applicability of PP mixtures within the investigated range of contamination, without much sacrifice in mechanical performance. However, in the case of ABS and PLA, more care should be taken when designing complex parts with weld lines, due to reduced toughness.

## 1. Introduction

Polymers, especially thermoplastics, play a vital role in modern times. They are extremely versatile in their use, such as packaging (39%), building and construction (23%), transportation (8%), electrical appliances and electronics (6%), and houseware (4%) [1,2]. Household plastics are made up of 15.4% polypropylene (PP), 13.4% low-density polyethylene (PE-LD), 9.1% polyvinyl chloride (PVC), 8.7% high-density polyethylene (PE-HD), 5.4% polystyrene (PS), and 5% polyethylene terephthalate (PET), in addition to other thermoplastics and thermosets [2]. When it comes to packaging, plastics should ideally not only provide the necessary protection for the product but also be easily recyclable, imposing a minimum impact on the environment [3].

Generally, plastics recycling follows one of three main approaches: thermal recycling (also termed as incineration), chemical recycling, and mechanical recycling (subdivided into primary and secondary recycling). Although we have come so far in recycling plastics, it is estimated that in Europe, only 18.5% of the European production is mechanically recycled [2], while the rest is incinerated or landfilled. In Germany, in 2021, 31.6% of total plastic waste was mechanically recycled while 58.3% was thermally recycled [4]. However, this does not even come close to the European circular economy target, pursuing the recycling of 50% of plastic waste by 2025 and 70% by 2030 [5,6]. On a global scale, the numbers are even more worrying, where globally 46% of the plastic waste ended up in landfills, 17% were incinerated, 22% were mismanaged and littered, while only 15% were collected for recycling in 2019, as per the OECD Global Plastic Outlook 2022 [7]. Based on that, the OECD estimates that only 9% were successfully recycled, taking recycling losses into consideration [8].

Chemical recycling is often the best option for highly diverse, contaminated, or deteriorated streams [9]. During pyrolysis, the hydrocarbon chains are broken, and the pure monomers or oils are recovered. These can be reused for the synthesis of new polymers, such as in the case of PVC [10], while having a lower emission and carbon footprint than incineration [11], in addition to preventing water and soil pollution, as in the case of landfilling [12]. Chemical recycling is expected to gain more importance in the future [13]. In contrast, incineration is relatively far down in the waste hierarchy and is, therefore, highly controversial. The process involves the burning off plastic waste and the recovery of energy [14].

Finally, mechanical recycling involves collecting, sorting, shredding, washing, drying, re-extruding, and pelletising the plastic. The thermal and mechanical stresses applied to plastics during mechanical recycling inevitably lead to material degradation [12,15]. The primary approach in mechanical recycling is mainly related to the recycling of uncontaminated waste of known history, such as post-industrial wastes. In this case, re-granulated and re-processed plastics [16] render properties comparable to those of virgin materials. Secondary recycling usually involves plastic streams of mixed types and colours of unknown history. Here, extensive sorting of the waste stream is needed to separate plastics according to their type [17].

Successful recycling of plastic waste requires an efficient sorting step ahead [18]. Macro-sorting is performed on whole or nearly whole objects, based on colour and material type, using optical and near-infrared sensors. The waste is then chopped into smaller flakes and further sorted according to polymer type and colour, using flake sorters. Further separation can be accomplished according to size, density, or electrostatic charging. For polymers with a large density difference, Dodbiba et al. [19] recommended the use of air tabbing, i.e., shaking the plastic flakes on a table with an inclined plane while jetting air. Light plastics are easily affected by air streams and thus move upwards, while heavier ones move downwards due to gravity. If the density difference allows for sink–float separation, then this process is preferred, as it renders high recovery rates [20]. In the case of small density differences, a tribo-electrostatic separation should be used.

Despite technological advances, the purity of plastic wastes is often limited to around 95% [17]. Separation of black or multilayer objects is still not possible; novel materials such as biopolymers are not yet accounted for. The contamination of recycling streams with unknown polymers can be extremely problematic. In the case of contamination with PVC, as an example, the generation of hydrochloric acid (HCl) during polymer processing can be extremely health hazardous and even further cause damage of the processing equipment, such as the barrels and the screws [21].

Generally, the influence of foreign polymers on the properties of recycled material is not yet clear. Elmaghor et al. [22] concluded that acrylonitrile butadiene styrene (ABS) can be easily dispersed in polycarbonate (PC) without a compatibilizer. Ruj et al. [23] claimed that up to 15% PVC in other polymers are not critical for low-grade products, while Serranti et al. [20] suggested that the purity of recycled material should be higher than 97% to be used in high-quality products. Low-grade recycling materials are often used in flooring tiles [24], gypsum boards to improve water absorption and swelling [25] or landscaping [20], concrete and asphalt [16], as well as in urban furniture. When reinforced polymers (e.g., with glass fibres) are processed with the same type of polymer, an increase in mechanical properties can be observed [26]. To improve the performance of recycled polymers, they are often mixed with virgin materials [27].

In recent years, the use of biopolymers has increased [16]. In 2022, global production of biopolymers reached 1.8 million metric tons [28], representing 1% of the total European plastic production [2]. They are considered especially favourable for packaging [3] due to their positive environmental impact [29]. However, until now, there has been no separate recycling stream for biopolymers, such as polylactic acid (PLA) [30]. Despite several benefits, biodegradable polymers can often only be composted under industrial conditions [31]. The contamination of recycling streams with bioplastics can thus be a larger issue in the upcoming years and can be very detrimental. Having 3% PLA in PET results in PLA agglomeration, whereas 5% PLA causes PET to stick to the walls of the mould during injection moulding [30]. In addition, the presence of 0.17% PLA causes transparent PET to become opaque [32,33]. On the other hand, Wojnowska-Baryła et al. [30] claimed that 10% of PLA does not have any significant effect on the mechanical properties of PS.

According to the above, recycling streams are often contaminated by foreign plastics due to sorting errors. The behaviour of polymer blends, however, is controversially discussed in the literature [20,21,22,23,24,25,26]. In this study, the effect of contamination of PP with some selected polymers as a result of mis-sorting on the mechanical behaviour is studied. PS and PET were selected based on their common use in packaging material and their frequent presence in the waste stream. ABS and PC were chosen as representatives for engineering polymers that are often incorrectly disposed in the packaging waste stream. PLA, one of the most commonly used biopolymers, is expected to find increased applications within the next couple of years and is not yet accounted for neither during sorting nor as a contaminant in other polymer streams.

## 2. Materials and Methods

The influence of PS, PET, ABS, PC, and PLA as a foreign polymer within PP (further referred to as the parent material) was investigated to assess the effect of mis-sorting during waste treatment on the quality and behaviour of recycled PP. For this purpose, different concentrations of the foreign polymer, ranging from 3% to 10%, were compounded with PP, pelletised, and further injection moulded to produce test samples. The samples were tested for mechanical behaviour.

### 2.1. Materials

Post-industrial PP, PS, PET, ABS, and PC were provided by Friedel Kunststoff Recycling GmbH in Böhmenkirch, Germany in the form of shredded flakes of around 3 mm in size. The post-industrial PLA used in this study was prepared in-house from yoghurt cup cut-outs, resulting from the thermoforming process.

It is worth mentioning that no datasheets were provided for the aforementioned post-industrial plastics. Therefore, the pure polymers were first characterised, and the results are summarised at the beginning of the Results section.

### 2.2. Sample Preparation

Material drying. Prior to extrusion, each polymer was dried in a Bierther DR 204 MT hot air dryer (Bad Kreuznach, Germany), with a dew point of −50 °C. ABS and PS were dried for 2 h at 80 °C, while PET and PC were dried for 5 h at 130 °C and 120 °C, respectively. PLA was dried overnight for at least 12 h at 50 °C.

**Compounding**. Different material mixtures were prepared by adding selected polymers (PS, PET, ABS, PC, PLA) to PP as the parent material at different gravimetric concentrations of 3%, 5%, 7%, and 10%. Material compounding was carried out on a Leistritz LSM 3034 twin screw co-rotating extrusion machine (Nürnberg, Germany), equipped with nine heating zones in addition to a separate heating zone for the die. The screws have a diameter of 34 mm with a length-to-diameter ratio of 30. Gravimetric dosing of the parent material and the supplement material was achieved using an MDS-Balance twin–feeder from Movacolor (VB Sneek, The Netherlands). Each feeder is equipped with a load cell that monitors the change in hopper mass over time and controls the screw speed of the feeder to ensure constant material feed and homogeneous mixture. The mixture was extruded into filaments of 2–3 mm diameter. Filaments were cooled in a 1.5 m water bath, dried, and pelletised. The pull speed of the pelletiser was kept constant for each mixture but had to be adjusted for each supplement to ensure adequate cooling, as fast speeds tend to shorten the polymer residence time in the cooling bath; insufficient cooling causes sticking problems within the pelletiser. The screw speed was kept constant at 147 rpm, and the throughput was set at 10 kg/h.

The temperature profile for compounding was selected as the minimum possible temperature that allows full melting of the foreign polymer, as detailed in Table 1. For all blends, the lie zone was set to be 5 °C cooler than the last extrusion heating zone.

**Injection moulding**. Before further processing, all mixtures were dried at 80 °C for 2 h. The test specimens were produced using a Demag 420/430 injection moulding machine (Schwaig, Germany), equipped with a single screw with three heating zones, in addition to a nozzle heater. Table 2 lists the injection moulding parameters for each case under consideration. The screw speed was set for all cases at 200 rpm.

Tensile specimens, according to DIN EN ISO 527-2, standard type 1A (10 mm width, 4 mm thickness) were injection moulded using a single film-type gate, with a longitudinal fill along the axis of the sample. Furthermore, a tensile sample (10 mm width, 3 mm thickness) with a weld line, in addition to tensile impact samples with and without weld line were produced. The weld line in the specimen’s cross-sectional area was obtained using a two-gate system at both ends of the cavity. The weld line forms a weak line in the middle of the sample and intensifies the contamination effect within the material. In addition, notched Charpy impact test specimens were injection moulded.

The mould was tempered at 40 °C for all cases. For each start of the machine or mould change, the mould was left to temper for at least 15 min; samples resulting from the first five injection moulding cycles were discarded to ensure equivalent conditions for all samples. For each material change, at least 300 cm³ of material was purged before injection moulding to avoid cross-contamination.

**Preparation of reference samples**. Further samples were similarly prepared from the pure polymers by extrusion, pelletising, and injection moulding. The main purpose was to determine the main characteristics due to the lack of a datasheet. Further, these samples served as reference samples during analysis and allowed for a better interpretation and understanding of the results. To avoid false conclusions based on different processing history, all pure polymers underwent the same processing steps as the blends.

Accordingly, the pure supplement materials were all processed at the same profile as that of its associated mixtures except for PC, which was processed at an increased temperature of 295 °C, since processing its mixtures at lower temperatures caused elevated torques on the machine.

Also, similar injection moulding conditions as those mentioned in Table 2 were applied for the pure polymers. Again, the mould was tempered at 40 °C for all cases, except for pure PLA, PC, and PET, where the mould temperature was set to be 30, 85, and 85 °C, respectively.

PP as the parent material was prepared at different temperatures corresponding to the processing temperature of the associated mix for reference purposes.

### 2.3. Material Testing

The main characteristics of the pure polymers and the influence of various foreign polymers on PP was quantified through the thermal, mechanical, and rheological properties of the material.

**Differential Scanning Calorimetry**. DSC was carried out on the pure and mixed polymers, using a Mettler Toledo calorimeter DSC 3+/Columbus (Ohio, USA). Samples of 10 ± 0.1 mg were introduced to an aluminium crucible of 0.4 µL capacity and further subjected to a first heating cycle to erase the thermal history of the materials, due to processing. After cooling, a second heating was carried out to determine the glass transition temperature T_g_ and melting temperature T_m_. An isotherm of 2 min was maintained between each heating/cooling cycle. Measurements were performed under a nitrogen atmosphere at a purge rate of 60 mL/min. For all materials under investigation, a heating/cooling rate of 20 K/min was applied. The start and end temperatures are listed in Table 3.

The glass transition temperature was determined as the midpoint temperature of the corresponding heat transition range. The melting temperature was identified at the peak of the exothermic reaction. The crystallinity was further calculated as per Equation (1), where ∆*H* is the heat adsorbed during the melting of the actual sample, which is determined by the area under the melting peak. ∆*H*_*crystal*_ is the enthalpy of melting a full crystal, i.e., the heat needed to melt the crystal per unit mass. ∆*H*_*crystal*_ for the main polymers under investigation were obtained from the literature [34]. In the case of the blends, ∆*H*_*crystal*_ of pure PP as the parent polymer was considered for calculation.

(1) $$X_{c}=\frac{∆H}{{∆H}_{crystal}}$$

**Tensile Test**. Tensile testing of samples with and without weld line was carried out using a Shimadzu Autograph AG-X plus universal testing machine (Kyoto, Japan), equipped with a 10 kN load cell and an optical extensometer (TRViewX). Tensile tests were performed according to DIN EN ISO 527-2 using tensile specimens with dimensions according to type 1A. A 20 N preload was applied to the specimen at the beginning of the test, followed by continuous loading at a crosshead speed of 1 mm/min up to 0.27% strain, to accurately determine the modulus of elasticity. After that, the test speed was increased to 50 mm/min up to failure. For all materials under consideration, at least 12 specimens were tested. The tensile modulus (E), yield strength ($\sigma_{y}$), and elongation at break ($\varepsilon_{b}$) were evaluated.

**Impact Test**. The impact strength of the materials under investigation in the tensile and bending modes according to DIN EN ISO 8256 and DIN EN ISO 179-1 (notched Charpy), respectively, was determined for specimens with and without the weld line.

Tensile impact testing was conducted on a Zwick-Roell mechanical impact tester (Ulm, Germany), equipped with a 7.5 J hammer for all materials, except for the pure polymer samples without a weld line, as well as the PC specimens with weld line; these required 50 J and 15 J hammers, respectively. In all cases, a traverse of a mass of 60.976 g was used. The energy at break $E_{c}$ was calculated according to the modified Equations (2)–(4) as per the standard, where $E_{s}$ is the measured absorbed impact energy, $E_{q}$ is the remaining energy in the pendulum after impact according to Equation (3), $m_{cr}$ is the mass of the traverse in kg, and $m_{p}$ is the pendulum mass in kg. The Charpy impact test of the single-notched specimens was performed with a 1 J hammer. The results of at least 12 specimens were considered.

(2) $$E_{c}=E_{s}-E_{q}$$

(3) $$E_{q}=\frac{E_{max}\times \mu \times \left(3+\mu \right)}{2\times \left(1+\mu \right)}$$

(4) $$\mu =\frac{m_{cr}}{m_{p}}$$

**Fourier-Transformation Infrared Spectroscopy**. The infrared (IR) spectra for all mixtures and pure materials under consideration were measured using the Shimadzu IRTracer-100 (Kyoto, Japan) in the mid infrared (MIR) range, using an attenuated total reflection (ATR), equipped with a diamond puck in the reflectance mode. The measurements were performed using a resolution of 4 cm^−1^, conducting 128 scans per spectrum to guarantee a high-resolution spectrum.

**Melt volume flow rate**. Injection moulded specimens were shredded to obtain small flakes of the different material blends under consideration. A Thermo HAAKE Meltfixer 2000 tester (Schwerte, Germany) was used to measure the melt volume flow rate (MVR) according to DIN EN ISO 1133-1 using the recommended test temperatures and weights for PP. The melt flow rate generally represents the speed of extrusion of a polymer under a defined temperature, through a defined die, and under a defined constant pressure. The tester chamber was thoroughly cleaned at the beginning of each measurement. Shredded material of 6 ± 0.05 g weight was introduced within 60 s into the chamber. After an initial compaction, achieved by a piston movement of 10 mm, the material was heated for 5 min. Then, the load was applied and the free fall piston travel per time was measured and further converted into the extruded volume per time. Ten measurements were taken at the 30 s interval, thus evidencing the effect of residence time on the flowability of the material. For each blend concentration, a total of three specimens would be tested.

**Statistical analysis**. ANOVA and Tukey HSD post hoc analysis was conducted using JMP^®^ Pro 17.0.0 to compare the effect of contamination concentration on the tensile properties and the MVR. Comparisons were made between the pure PP and the different contaminations with ABS, PC, PET, PLA, and PS at 3%, 5%, 7%, and 10%.

## 3. Results

### 3.1. Main Characteristics of Post-Industrial Plastics

The pure polymers under consideration were tested for their thermal and mechanical properties, as well as for their MVR. Results are summarised in form of a brief datasheet in Table 4. The mechanical properties are related to samples without parting lines.

Moreover, samples of the parent polymer PP were prepared at different temperatures corresponding to the processing of its blend with the specific supplement polymer to eliminate the effect of processing temperature when comparing the behaviour of the blends with the pure polymer. These results are further indicated in Table 4.

### 3.2. Effect of Foreign Polymer Inclusion on the Tensile Behaviour of PP

**Figure 1:**
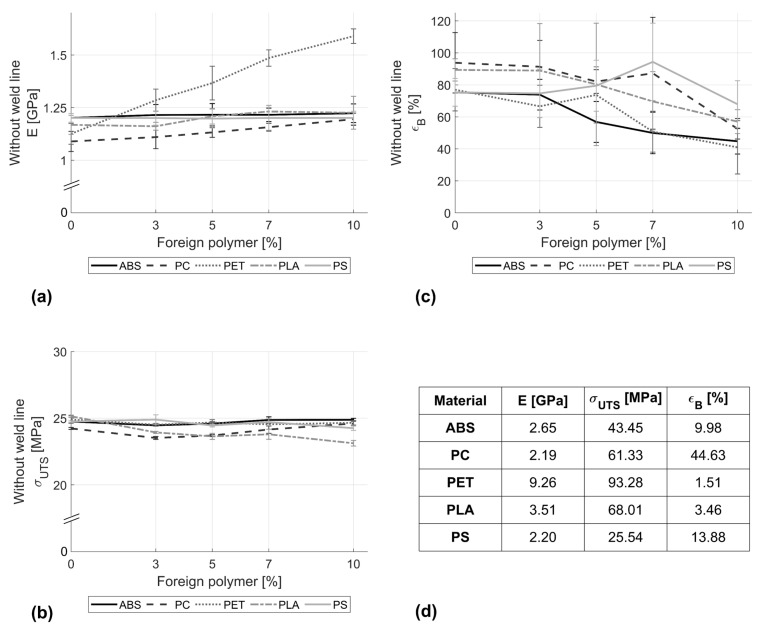
Effects of adding ABS, PC, PET, PLA, and PS on the (**a**) stiffness, (**b**) ultimate tensile strength, and (**c**) elongation at break of PP stream in the absence of a weld line. (**d**) Reference values of pure supplement polymers.

Figure 1 shows the effect of adding ABS, PC, PET, PLA, and PS on the tensile properties of the parent PP material. It can be observed that the addition of ABS and PS up to 10% has no significant impact on the tensile modulus at a confidence level of 95%. In contrast, there was a significant difference in the tensile modulus among the different PET contamination percentages F(4, 55) = 123.25, *p* < 0.0001. Post hoc results revealed significant differences in tensile modulus between all concentrations under investigation. While in the case of the 10% PP/PC mix, the modulus of elasticity increased by 9.7% compared to pure PP, the addition of 10% PET to the PP stream resulted in an increase of 41.2% in the tensile modulus.

In most cases, contamination at low percentages of 3% showed a significant reduction in the tensile strength compared to the parent polymer, according to the results of the statistical analysis. However, higher percentages do not cause further significant changes in the mean value M of the tensile strength, as in the case of PET, for example, with M = 24.51, SD = 0.09 at 3% contamination with respect to M = 24.66, and SD = 0.17 in the case of 10% PET.

A maximum reduction in the elongation of break of 46.8% was observed in the case of the PP blend with 10% PET. It is also worth noting that the inclusion of up to 7% PS was found to increase the elongation at break by 25.3%, as well as the associated scattering. Accordingly, the ANOVA revealed no significant changes in the elongation at break in the case of contamination of PP with PS and PLA (*p* > 0.05). In the case of PC, the Tukey HSD showed that only the 10% concentration had a significantly lower elongation at break (M = 54.18, SD = 8.83) compared to the parent polymer (M = 107.17, SD = 53.70).

**Figure 2:**
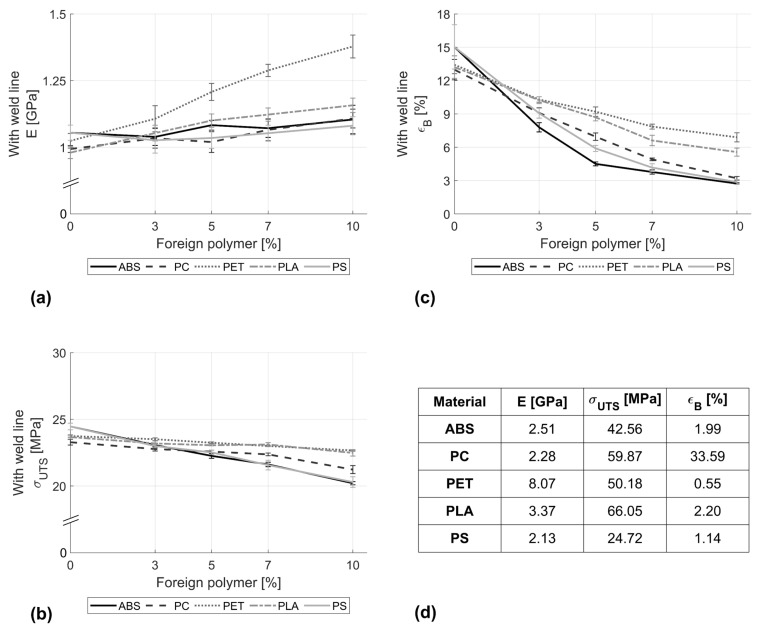
Effects of adding ABS, PC, PET, PLA, and PS on the (**a**) stiffness, (**b**) ultimate tensile strength, and (**c**) break elongation of PP stream in the presence of a weld line. (**d**) Reference values of pure supplement polymers.

Figure 2 shows the effect of the polymer inclusions on the tensile modulus, strength, and elongation at break of PP when a weld line is present in the specimen. It can be seen that supplements of up to 10% barely influence the stiffness of PP. This can be related to the fact that the tensile modulus is measured within the elastic region, where the weld line does not yet have much influence. However, in the case of the PET as an inclusion within PP, an increase of 34.5% in the tensile modulus of PP was observed.

In contrast, the tensile strength was found to suffer from the presence of foreign polymers within the PP. The inclusion of 10% ABS or PS resulted in a 17.5% reduction in strength. In the case of PP mixtures with PET or PLA, only minor changes (<5%) in tensile strength could be observed. The elongation at break was largely affected by foreign inclusions, even at minor concentrations of 3%. The elongation at break was found to decrease with increasing amounts of foreign polymer within the parent material. At 10% ABS, a loss of more than 80% in the elongation at break with respect to the pure PP could be detected.

On the basis of the aforementioned results, it becomes evident that the presence of a weld line emphasises the effect of foreign polymers within PP. In this work, weld lines were intentionally formed during injection moulding, creating two separate melt streams that meet at the centre of the mould cavity. Due to the fact that these streams take different paths, temperature differences are more likely to occur between the two, leading to stress concentrations [35,36,37], discontinuity in macromolecular entanglement [37], and discontinuous macromolecular orientation parallel to the weld line [38]. In all cases, the samples under investigation failed due to this artificially induced defect, leading to a loss of mechanical properties. In specimens without the weld line, material failure would occur at the weakest point of statistically distributed molecular defects. In the presence of foreign polymer, these often act as inclusions and are related to higher stress concentrations, and thus form sites for failure initiation. This effect can be seen for concentrations higher than 5%.

### 3.3. Effect of Foreign Polymer Inclusion on the Impact Behaviour of PP

**Figure 3:**
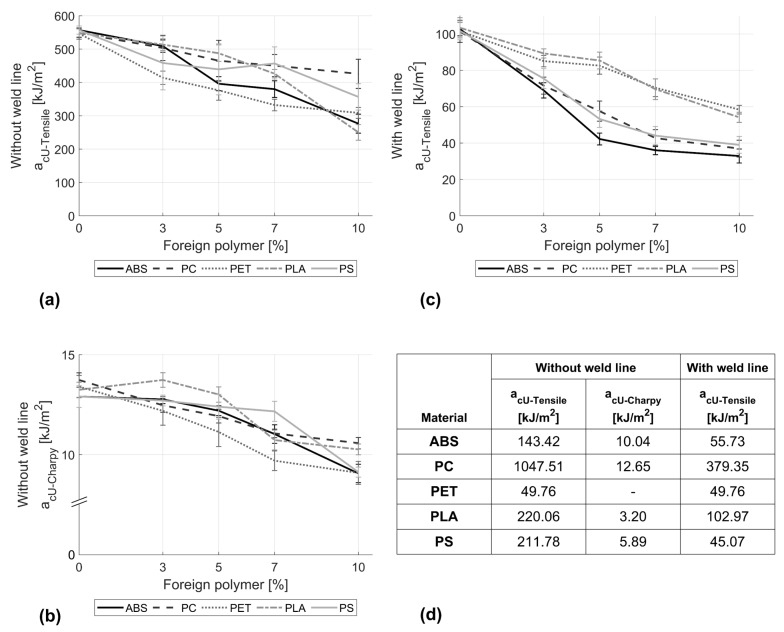
Effects of adding ABS, PC, PET, PLA, and PS on the impact strength of PP in (**a**) tensile and (**b**) bending (Charpy) mode in the absence of a weld line, as well as (**c**) in tensile mode in the presence of a weld line. (**d**) Reference values of pure supplement polymers.

Figure 3a,b show the impact test results carried out on samples without a weld line in tensile and bending modes. Regardless of the test type, the inclusion of foreign polymers resulted in a clear reduction in the impact strength of PP. This deterioration increases with increasing concentration of the foreign polymer. However, the trend is not the same for all supplement polymers under consideration. As depicted in Figure 3b, the Charpy impact strength of PP mixed with up to 7% PS remains unaffected, in contrast to the impact strength of pure PP, as also evidenced by the post hoc analysis, which indicates no significant changes in the Charpy impact strength between 0%, 3%, and 5% PET. It is first at 10% PS within the mix that a significant reduction in the mean impact strength of M = 9.17, SD = 0.33 in contrast to M = 12.9, SD = 0.54 for the pure PP can be truly claimed. In the case of ABS at 3% within ABS, no significant effects on the impact strength could be detected. However further contaminations evoke significant reductions, reaching a loss of nearly 30% in toughness at 10% ABS within PP. Again, it is the PET that shows the most severe changes among all supplement materials under consideration. Here, statistical analysis proves significant reduction in the impact strength, even at low concentrations. At higher percentages of 7% and 10%, the toughness values seem to first stabilise showing no significant differences in the mean values (M = 9.8, SD = 0.63 vs. M = 9.09, SD = 0.57, respectively).

A similar trend was observed in the case of the tensile impact test (Figure 3a), although the results were an order of magnitude higher than those obtained in the bending mode. This can mainly be related to the sample configuration and the type of load. In the case of the Charpy impact test, the bending load causes tensile stresses at the notch area, resulting in crack initiation and further propagation at low energy absorption levels across the cross section of the sample. However, impact tension induces tensile stresses throughout the cross section of the sample, requiring higher energy up to failure. It should be noted that it was not possible to test the tensile impact of pure PET specimens, as these tended to slip out of the grips; an increase in clamping force inevitably led to material crushing.

Figure 3c shows the effect of foreign polymers on the tensile impact strength of PP in the presence of a weld line. Similar to the aforementioned observation in the case of samples without a weld line (Figure 3b), the tensile impact strength of PP was significantly reduced even at low concentrations of 3% of the supplement polymer. An increase in the percentage of SPs within PP leads to a continuous reduction in the tensile impact strength. At concentrations of 10% ABS, PS, or PC, PP was found to retain only around 40% of its impact strength. In contrast, PET and PLA inclusions appeared to have lower impacts, on average retaining 55% of the initial impact tensile strength.

### 3.4. Effect of Foreign Polymer on the Melt Volume Flow Rate of PP

**Figure 4:**
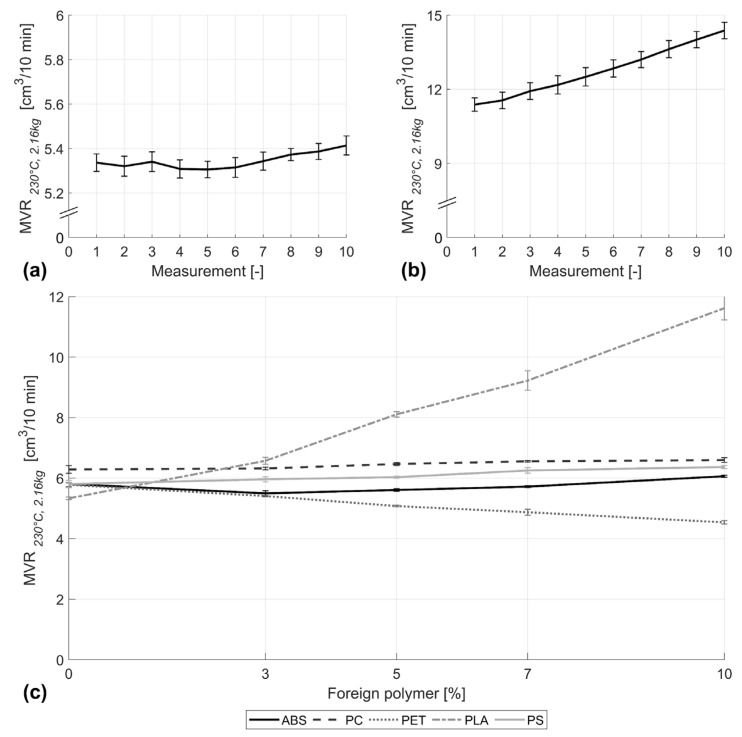
Melt volume flow rate for (**a**) pure PP processed under the same conditions as for the PP/PLA blend, (**b**) PP with 10% PLA, and (**c**) PP and its blends.

Figure 4a depicts the average measurements carried out for the case of pure PP. Results indicate insignificant changes in MVR over time at small standard deviation. In contrast, a PP blend including 10% PLA (usually processed at temperatures lower than 230 °C) rapidly degrades, leading to an increase in MVR from 11.3 cm^3^/10 min to 14.3 cm^3^/10 min within 10 min, as per Figure 4b. To ensure the comparability of measurements, the average MVR was calculated for all blends under consideration using only the first three measurements.

Based on that, Figure 4c shows the change in MVR of PP for all blends under consideration. Among all the contaminating materials, PLA seems to have the greatest influence on the MVR, causing a significant increase of 118% at a loading percent of 10. ANOVA and post hoc analysis further proved a significant increase in the MVR with each increase in PLA concentration. In contrast, the contamination of PP with PET showed a steady but significant (*p* < 0.0001) reduction in the MVR, reaching 21% at 10% PET, which relates to an increased viscosity. PC, PS, and ABS showed comparatively little impact on the MVR, where an increase averaging 3.5%, 8.6%, and 4.5%, respectively, could be detected. However, it should be noted that for 3% ABS in PP, the MVR of PP first witnesses a slight initial significant decrease of 4.4% (M = 5.8, SD = 0.09) with respect to the pure PP (M = 5.8, SD = 0.12) before increasing once more with further increase in the ABS content.

### 3.5. Effect of Foreign Polymer Inclusion on the IR-Spectrum of PP

**Figure 5:**
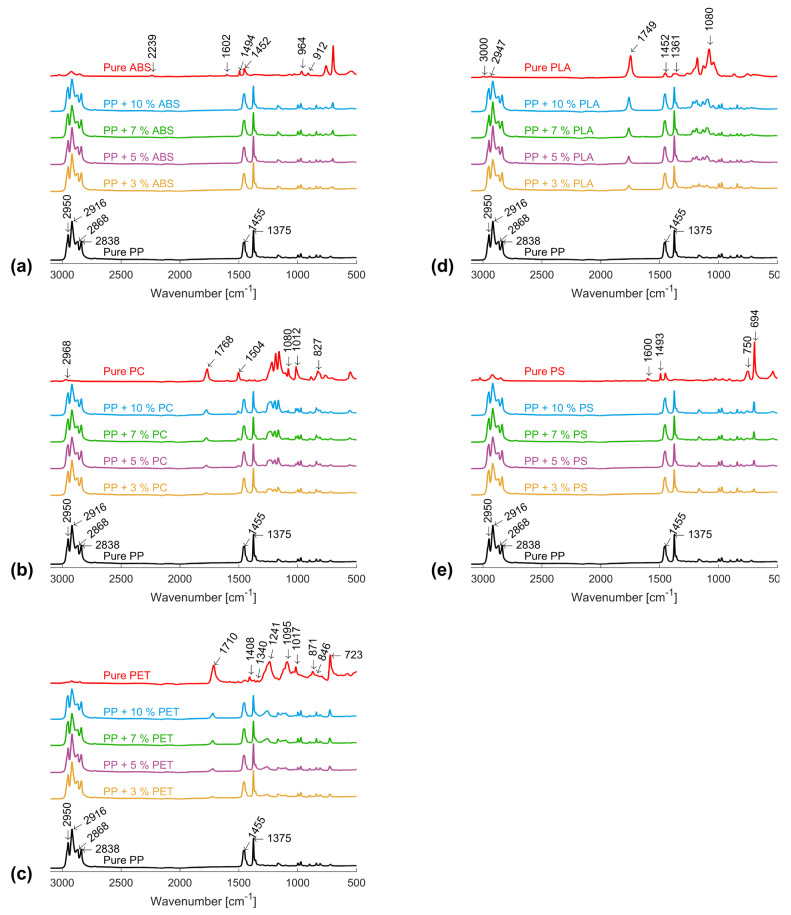
FTIR spectrum of (**a**) PP, ABS, and their blends; (**b**) PP, PC, and their blends; (**c**) PP, PET, and their blends; (**d**) PP, PLA, and their blends; and (**e**) PP, PS, and their blends.

Figure 5a shows the results of the FTIR scan of the PP and its mixtures with ABS. The distinctive peaks of PP provide evidence of the symmetric and asymmetric stretching of the CH_3_ group at 2868 cm^−1^ and 2950 cm^−1^, respectively, as well as those of the CH_2_ group at 2838 cm^−1^ and 2916 cm^−1^, respectively. The CH_3_ vibration in umbrella mode at 1375 cm^−1^ and that of the symmetric in-plane vibration of the C-H(CH_3_) group at 1455 cm^−1^ were also easily identified in the pure PP spectrum and are in line with the literature findings [39,40].

The IR spectrum of the pure ABS under consideration shows the characteristic absorbance peaks of the acrylonitrile C≡N bond at 2239 cm^−1^ and the styrene’s aromatic ring vibration at 1602 cm^−1^ and 1494 cm^−1^, the scissoring of the CH_2_ group at 1452 cm^−1^, and the C-H deformation of the hydrogen atoms found in the alkenic carbon in the butadiene phase at 964 cm^−1^ and 912 cm^−1^, all of which are in accordance with the literature [41,42]. The mixture of PP and ABS evidences the existence of the distinctive peaks of both polymers.

The spectra of PC and its blends with PP are illustrated in Figure 5b. The characteristic vibration peaks of PC, including the asymmetric stretching of the C-H group at 2968 cm^−1^, the C=O stretching at 1768 cm^−1^, the C-C stretching at 1504 cm^−1^, the C-C-C bending at 1080 cm^−1^, the O-C-O simultaneous stretching at 1012 cm^−1^, and the C-H deformation at 827 cm^−1^ could be clearly identified [39,42].

The effect of mixing different concentrations of PET in PP on the IR-spectrum of PP is summarised in Figure 5c. Numerous distinct PET peaks, in accordance with the literature [39], could be identified, including the aromatic skeletal stretching band at 1408 cm^−1^; the CH_2_ wagging at 1340 cm^−1^; the C=O stretching at 1710 cm^−1^; the broad peaks at 1095 cm^−1^ and 1241 cm^−1^ due to ester stretching; the benzine ring vibration at 871 cm^−1^, 723 cm^−1^, and 1017 cm^−1^; and the CH_2_ rocking at 846 cm^−1^. Here, it is the peak at 1710 cm^−1^ that showed a varying intensity with the PET concentration in PP.

In the case of mixing PLA into PP, Figure 5d shows the existence of the characteristic stretching of C-O bonds at 1080 cm^−1^, C=O stretching at 1749 cm^−1^, the symmetric and asymmetric stretching of CH_3_ at 2947 cm^−1^ and 3000 cm^−1^, as well as their symmetric and asymmetric bending at 1361 cm^−1^ and 1452 cm^−1^. Here, the intensity of the C=O stretching at 1749 cm^−1^ varies with the concentration of PLA in PP [43].

Finally, Figure 5e illustrates the IR spectra of PP and its mixtures with PS. According to [39], the distinctive benzine ring vibration peaks lie at 1600 cm^−1^ and 1493 cm^−1^. The out-of-plane mono-substitution deformation peaks at 694 cm^−1^ and 750 cm^−1^ of PS changes its intensity with the varying content of PS within PP. Table 5 summarises the distinctive identification wavenumbers and their associated bonds for each of the tested polymers.

### 3.6. Effect of Foreign Polymer Inclusion on Glass Transition and Melting

Table 6 summarises the glass transition and melting temperatures for the pure polymers and the polymer blends under investigation. Further, the heat absorbed during melting *ΔH*, the heat needed to melt a full crystal *ΔH_crystal_*, and the calculated crystallinity *X_c_* are presented in the table.

Considering the crystallinity of the materials under investigation, it can be observed that the contamination of PP with the foreign polymers under investigation generally caused a slight reduction in crystallinity of PP ranging between 11% (in the case of PP/PET) and 23% (in the case of PP/PC). However, no clear trend can be depicted, except in the case of PLA, where the increase in PLA concentration within PP resulted in an increase in crystallinity. However, the initial value of 47.5% related to pure PP could not be exceeded, even at 10% PLA.

It can be noted that the glass transition and melting temperatures of the blends were highly dependent on the parent polymer PP. Accordingly, in the case of the amorphous supplement polymers ABS, PC, and PS, a melting temperature of around 152 °C was detected. Hence, the contamination of PP by either of these two polymers reduced the melting temperature of PP by around 7%. A similar trend could also be observed in the case of the semi-crystalline supplement materials. These results support the hypothesis that the contaminating polymers remain incorporated as inclusions within the parent polymers and do not interact with it, as evidenced by the FTIR results.

**Figure 6:**
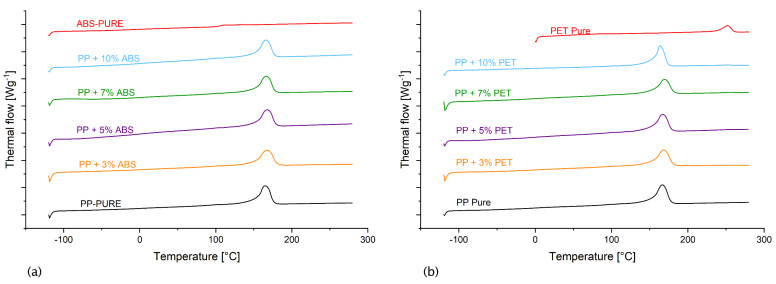
DSC curves of (**a**) PP, ABS, and their blends; and (**b**) PP, PET, and their blends.

Figure 6 shows the results two exemplary DSC investigations. Figure 6a shows the behaviour of the PP/ABS blend (amorphous supplement polymer) with respect to the heat flow of pure PP and pure ABS. It can be easily depicted that the thermal behaviour of the blend is dominated by the behaviour of the pure PP. The melting temperature is minimally shifted as described above, while the T_g_ of the pure ABS diminishes. Similarly, in the case of the semi-crystalline supplement polymer PET (Figure 6b), the melting temperature of pure PET at 252 °C can be depicted. However, the blends do not show any reaction at this elevated temperature. In contrast, they show a melting peak in the range of 150 °C, corresponding with the melting point of pure PP.

## 4. Discussion

**ABS as a foreign polymer in the PP recycling stream.** In the case of a good bond between PP and ABS within the blend, elastomeric polybutadiene in ABS [44] is expected to contribute towards improved toughness of the semi-crystalline PP. However, such an interface is only achievable through the addition of compatibilisers [26,45,46]. The current study confirms these findings, as the incorporation of ABS into PP without such coupling agents did not notably affect the tensile stiffness or strength of the parent polymer. Even at higher concentrations of 10% generally. For concentrations of up to 3%, no significant effect on the ductility of PP could be observed with respect to the parent polymer. This is also supported by the MVR results, which suggest that for lower ABS concentrations, no structural changes occurred that might have led to a change in the flowability of PP.

Based on these results, up to 3% ABS can be allowed within a PP stream without significantly affecting its mechanical and rheological properties negatively. Due to the acceptable tensile properties observed in samples with a weld line, it can be assumed that such a recycling material can be used in complex products that may require multiple gates during injection moulding. For simpler parts (without weld lines), lower-quality streams with 5–10% ABS can be used without any sacrifice in strength, stiffness, or processability of PP.

**PC as a foreign polymer in the PP recycling stream.** PC is an amorphous polymer, which is immiscible with PP [47,48,49,50,51]. Due to the high melting temperature of PC (280–315 °C) with respect to that of PP (215–260 °C), the mixture was processed at the higher temperature levels to ensure a full melt of the PC. However, at 280 °C, PP showed a 2.14% reduction in tensile strength, compared to PP processed at 245 °C (as in the case of the experimental setup for the ABS blend). A similar observation was reported by Van Bruggen et al. [52], where the impact strength of pure PP decreased by 3.7%, when the processing temperature of pure PP was increased from 250 °C to 300 °C.

Despite the deterioration in strength of PP under the given processing conditions and the immiscibility of PC and PP, the mix did not show a notable loss in strength and stiffness. This can be interpreted in terms of the good dispersion of the PC phase during the compounding phase. As a result, the well-dispersed PC phase associated with higher mechanical properties acts as a reinforcement within the PP, thus retaining its overall mechanical performance.

According to these findings, for parts produced without a weld line, contamination of PP with PC of up to 5% will not have a significant effect on the mechanical properties of PP, providing the mix is compounded at temperatures high enough to melt the contaminating polymer. Once compounded, the mixture can be injection moulded at the conventional processing temperatures of PP without significant change in the MVR of PP. The PC would then act as a dispersed strengthening phase. However, for applications with weld lines, a decrease in toughness and elongation at break should be expected, even at low contaminations of 3%.

**PET as a foreign polymer in the PP recycling stream.** PET is immiscible in PP [53,54] due to the lack of chemical and polar compatibility. PP/PET blends tend to establish a two-phase morphology, where relatively large spherical droplets of PET are present within PP [55,56,57]. This theory is supported by the MVR findings, indicating a significantly increased viscosity of the PP/PET blend, with respect to the parent polymer, since the flow of PP is hindered by the crystalline PET phases. Compatibilizers are necessary to create a bond between the two phases. The results presented here show that PP/PET blends behaved according to the rule of mixtures, where the stiffness of the mix linearly increased with increasing PET content, while the ductility linearly decreased. Here, PET acts as the stiffening component to the more ductile PP. Similar results were reported in [58,59,60,61,62], where fabrics and textiles produced out of a polyolefin/PET blend showed superior properties. Li et al. [63] claimed that the inclusion of high-melting-temperature polymers into PP acts as a microfibrillar reinforcement. In addition, Inoya et al. [64] proposed that a homogeneous PP/PET blend can be achieved by deformation of the dispersed phase through adequate mixing to prevent its agglomeration.

The present study proves that a 3% mis-sorting of PET already results in significant property changes of the PP, where a stiffness increase of 15% is accompanied by a 6.8% and 9% drop in elongation at break and Charpy impact strength, respectively. At higher concentrations of PET, recycled material can further be considered for complex parts involving a weld line, as it behaves as a reinforced PP blend with enhanced stiffness. However, care should be taken during processing, since higher injection pressures are to be expected due to increased melt viscosity (MVR increase by 21% at 10% PET).

**PLA as a foreign polymer in the PP recycling stream.** Similar to the other polymers under consideration in this study, PLA is also incompatible with PP. Consequently, PP/PLA blends tend to form a multiphase system with poor mechanical performance [65,66,67]. PP-g-maleic anhydride is often used as a compatibilising agent to mitigate the blend’s polarity for a better interface. Whereas small amounts of 3% PLA within PP already caused significant reduction in tensile and impact strength as well as in the MVR, stiffness was first significantly affected at a concentration of 10%.

Based on the above results, it is recommended to use PP streams with PLA mis-sorting in applications that do not require high toughness or complex gating systems that lead to the formation of weld lines. Such a mix is, however, still usable in general applications where stiffness is the key factor. It should be noted that in this study, the PP stream with PLA mis-sorts was processed at 215 °C, which is normally considered too low for PP (225–245 °C). Processing at higher temperatures around 240 °C cause PLA degradation (as evidenced by the significantly increased MVR), which could lead to lower ductility and toughness of the mix. It is to be noted that the reduced viscosity must also be considered at the processing stage.

**PS as a foreign polymer in the PP recycling stream.** PS is immiscible in PP [68]. Therefore, it is generally suggested to use compatibilisers for PP/PS blends. However, in [69,70], high shear mixing techniques were used to efficiently blend PP and PS without the addition of compatibilisers. Here, a twin-screw counter-rotating extruder was used to bring high shear into the material. Although this is generally not the case in this study, the incorporation of PS into PP did not negatively influence the stiffness and tensile strength or its processability. For up to 5% PS, there was even no significant change in ductility or toughness with respect to the parent polymer. A further increase in PS content to 7% seemed to improve ductility, although it was not statistically significant. This effect is nullified at a higher PS content of 10%, bringing back the ductility to its original value, while having a negative impact on the toughness.

Experiments carried out on samples with a weld line evidence that the incorporation of PS hinders good bonding at the weld line, thus linearly decreasing the overall toughness, ductility, and tensile strength with increasing PS content. On the one hand, the benzine ring within the PS chain is expected to hinder the mobility of the PP molecules, thus strengthening the material. However, the immiscibility of PP and PS encourages the agglomeration of PS, thus decreasing overall mechanical performance. These two mechanisms work against each other, which is reflected in the increased standard deviation of the elongation at break for samples without a weld line up to 7% PS. A further increase in the PS content is expected to encourage the formation of agglomerates, leading to the deterioration of the ductility. Hence, it can be concluded that the mis-sorting of PS up to 7% will not cause a significant change in the mechanical properties of PP. At 10% PS, only the toughness of PP would suffer. If this mix is, however, used in the manufacturing of complex geometries that require multiple gates in the mould, a significant decline in the ductility and toughness of PP should be expected. However, this should not render this polymer stream unusable for mechanical recycling.

**Figure 7:**
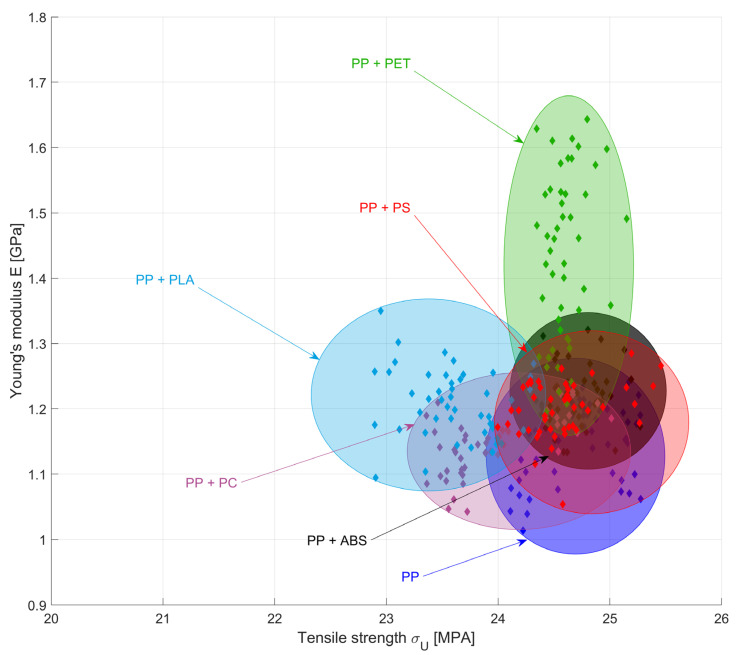
Effects of supplement polymer inclusion on the strength and stiffness of PP.

Figure 7 provides a graphical summary of the results of this study. The plot depicts the properties of PP when mixed with up to 10% of the foreign polymer.

## 5. Conclusions

The contamination of polypropylene with selected polymers is studied to simulate the effect of mis-sorting in recycling streams. ABS, PC, PET, PLA, and PS are all immiscible in PP. Conscious mixing was studied in the literature and usually involved the addition of coupling agents to improve bonding between PP and the supplement material. However, there are no clear and systematic information related to the non-intentional contamination of PP by other plastics. PP constitutes one of the largest fractions of plastic recycling streams with great potential for re-integration in new applications, especially under the limelight of new regulatory aspects.

This study proves that to some extent the presence of foreign polymers in PP can be accepted. Accordingly, it is generally recommended to compound the blend at temperatures high enough to ensure the melt of the supplement polymer by using a twin-screw extruder before injection moulding the final product. Many of these contaminants then tend to form a secondary reinforcing phase within the PP.

It can be concluded that the inclusion of PS and ABS has no significant impact on the tensile modulus; the addition of PLA and PC shows significant impacts on stiffness first at higher concentrations of 10% and 7%, respectively. In contrast, the tensile modulus is significantly affected by the addition of PET, even at low concentrations of 3%. The tensile strength, however, proved to be significantly affected by the addition of any of the supplement polymers under consideration.

Moreover, it can be stated that ABS, PC, and PS inclusions, although not affecting its processability, generally render PP unsuitable for complex parts that involve a weld line due to reduced toughness and ductility. However, such mis-sorts are acceptable for simple components without a weld line. Inclusion of PET or PLA within PP at 10% would generally result in reduced toughness and ductility. The introduction of a weld line emphasised the effect of the defects within the PP blend. Following these results is generally the more conservative way for product design. In accordance, knock-down or safety factors can be reduced.

Moreover, PLA significantly degrades at dwell times greater than 3 min, leading to an increased MVR of 118% after 10 min. This might render the PP stream unsuitable for extrusion applications. Amongst all supplement polymers under investigation, PET was the only one causing a decrease in the MVR of PP (by 22%). Further investigations proved that contamination of PP with foreign polymers resulted in a slight reduction in crystallinity of PP ranging between 11% in the case of PET and 23% in the case of PC.

It is to be noted that the study was limited to only one sort of PP and contaminating plastics, originally resulting from post-industrial recyclates. The use of other plastic types will expectedly lead to other values and other application ranges. Extended studies, eventually linked to artificial intelligence algorithms, can lead in future to a better understanding of the behaviour and assist in the identification of suitable processing windows.

Finally, care must be taken that for some applications, there are often further material demands, other than the thermal and mechanical properties. In the case of food packaging, as an example, certain levels of purity must be met. Also, regulations require food-grade materials for use in the food sector. Here, future developments in the recycling field should not only focus on improved waste sorting, based on the polymer type, but further improve technologies to differentiate between food and non-food grades within the same polymer type.

## Figures and Tables

**Table 1:** Material extrusion parameters.

		Polymer Blends				
		ABS	PS	PET	PLA	PC
**Temperature [°C]**	Zone 1	160	160	170	130	195
	Zone 2	210	210	220	180	245
	Zone 3	220	220	230	190	255
	Zone 4	225	225	235	195	260
	Zone 5	230	230	240	200	265
	Zone 6	230	230	240	200	265
	Zone 7	240	240	250	210	275
	Zone 8	240	240	250	210	275
	Zone 9	245	245	255	215	280
	Die zone	240	240	250	210	275
**Screw speed**		147 rpm				
**Throughput**		10 kg/h				

**Table 2:** Injection moulding parameters.

Polymer Blends						
		ABS	PS	PET	PLA	PC
**Temperature [°C]**	Zone 1	195	195	195	195	195
	Zone 2	205	205	205	205	205
	Zone 3	210	210	210	210	210
	Nozzle	220	220	220	220	220
	Mould	40	40	40	40	40
	Drying	80	80	80	80	80
**Drying time [h]**		2	2	2	2	2
**Screw speed [mm/s]**		200				
**Injection pressure [bar]**		▪ Tensile specimens without weld line: 500 ▪ Tensile specimens with weld line: 480 ▪ Specimens for Charpy-impact testing: 325				
**Holding pressure [bar]**		▪ Tensile specimens without weld line: 300 ▪ Tensile specimens with weld line: 300 ▪ Specimens for impact testing: 210				
**Holding time [s]**		▪ Tensile specimens without weld line: 12 ▪ Tensile specimens with weld line: 12 ▪ Specimens for impact testing: 10				
**Cooling time [s]**		15				

**Table 3:** Start and end temperatures for DSC measurements for the different pure polymers and polymer blends.

Polymer	Starting Temperature [°C]	End Temperature [°C]
**Pure PP, pure ABS,** **PP-PLA, PP-ABS, PP-PC, PP-PS, PP-PET**	−120	280
**Pure PET, PURE PC**	0	300
**Pure PLA**	0	210
**Pure PS**	0	280

**Table 4:** Brief datasheet of the pure polymers, as a result of their own characterization.

	PP (@275 °C)	PP (@210 °C)	PP (@250 °C)	PP (@240 °C)	ABS	PS	PET	PLA	PC
**σ_UTS_ [MPa]**	24.2 ± 0.1	25.1 ± 0.1	24.9 ± 0.3	24.8 ± 0.1	43.5 ± 0.1	25.5 ± 0.4	93.3 ± 1.6	68 ± 0.6	61.4 ± 0.2
**E [MPa]**	1089 ± 48	1190 ± 95	1126 ± 50	1209 ± 34	2646 ± 59	2197 ± 62	9259 ± 532	3507 ± 104	2193 ± 133
**ε_B_ [%]**	107.2 ± 53.7	105.1 ± 59.8	77 ± 13.3	77.3 ± 11.3	10 ± 2.70	13.9 ± 5	1.5 ± 0.1	3.5 ± 0.5	44.6 ± 22.5
**a_cU-Tensile_ [kJ/m^2^]**	548.1 ± 13.4	548.4 ± 12	546.7 ± 17.1	557.1 ± 12.3	143.4 ± 20	211.8 ± 32	49.8 ± 3.7	220.1 ± 18	1047.5 ± 206
**a_cU-Charpy_ [kJ/m^2^]**	13.7 ± 0.4	13.2 ± 0.4	13.4 ± 0.6	12.9 ± 0.5	10 ± 0.3	5.9 ± 0.6	N/A	3.2 ± 0.2	12.7 ± 0.5
**T_g_ [°C]**	not clearly detectable				104.1	90.7	74.5	88.9	170.3
**T_m_ [°C]**	163.3	166.6	166.7	165.1	N/A	N/A	252.1	210.3	N/A
**ΔH_m_ [J/g]**	101.8	98.4	101.8	94.4	N/A	N/A	24.1	48.4	N/A
**MVR** **[cm³/10 min]** **(temp/load)**	6.3 ± 0.1 (230/2.16)	5.3 ± 0.1 (230/2.16)	5.8 ± 0.1 (230/2.16)	5.8 ± 0.1 (230/2.16)	35.8 ± 0.4 (220/10)	17.8 ± 0.2 (200/5)	36.7 ± 0.6 (280/2.16)	183.4 ± 0.2 (210/2.16)	75.5 ± 36.2 (300/1.2)

**Table 5:** Distinctive FTIR wavenumbers associated with polymer identification [39,40,41,42,43].

Wavenumber [cm^−1^]	Assignment	ABS	PC	PET	PLA	PP	PS
694	Out-of-plane mono-substitution						●
750	Out-of-plane mono-substitution						●
723	Benzine ring vibration			●			
827	C-H deformation		●				
846	CH_2_ rocking			●			
871	Benzine ring vibration			●			
912	C-H deformation in the butadiene phase	●					
964	C-H deformation in the butadiene phase	●					
1012	O-C-O simultaneous stretching		●				
1017	Benzine ring vibration			●			
1080	C-C-C bending		●				
1080	C-O bond stretching				●		
1095	Ester group stretching associated with saturated ester			●			
1241	Ester group stretching associated with ethyl acetate			●			
1340	CH_2_ wagging			●			
1361	Symmetric bending of CH_3_ group				●		
1375	Umbrella vibration of CH_3_ group					●	
1408	Aromatic skeletal stretching			●			
1452	Scissoring of CH_2_ group	●					
1452	Asymmetric bending of CH_3_ group				●		
1455	Symmetric in-plane vibration of C-H(CH_3_)					●	
1493	Benzine ring vibration						●
1494	Aromatic styrene ring vibration	●					
1504	C-C ring stretching		●				
1600	Benzine ring vibration						●
1602	Aromatic styrene ring vibration	●					
1710	C=O stretching of aromatic carboxylic acid ester			●			
1749	C=O stretching of saturated aliphatic ester				●		
1768	C=O stretching of carboxylic acid phenyl ester		●				
2239	Acrylonitrile C≡N bond	●					
2838	Symmetric stretching of CH_2_ group					●	
2868	Symmetric stretching of CH_3_ group					●	
2916	Asymmetric stretching of CH_2_ group					●	
2947	Symmetric stretching of CH_3_ group				●		
2950	Asymmetric stretching of CH_3_ group					●	
2968	Asymmetric C-H group stretching		●				
3000	Asymmetric stretching of CH_3_ group				●		

**Table 6:** Glass transition and melting temperatures as well as melting enthalpy [34] and crystallinity measured by DSC for the different pure polymers and blends under consideration.

	T_g_ [°C]	T_m_ [°C]	ΔH_m_ [J/g]	ΔH_crystal_ [J/g]	X_c_ [%]
**PP-ABS00 (@240 °C)**		165.05	94.44	207	45.62
**PP-ABS03**	−0.53	152.23	78.49	207	37.92
**PP-ABS05**	3.81	153.87	79.73	207	38.52
**PP-ABS07**	1.97	153.02	78.08	207	37.72
**PP-ABS10**	−6.24	153.07	77.91	207	37.64
**ABS_PURE**	104.13				
**PP-PC00- (@275 °C)**		163.27	101.76	207	49.16
**PP-PC03**	−1.65	151.87	78.32	207	37.83
**PP-PC05**	−6.85	152.44	74.71	207	36.09
**PP-PC07**	7.23	152.59	78.64	207	37.99
**PP-PC10**	−2.55	152.99	78.29	207	37.82
**PC_PURE**	170.34				
**PP-PET00 (@250 °C)**		166.68	101.81	207	49.18
**PP-PET03**	5.08	153.97	79.37	207	38.34
**PP-PET05**	5.18	153.64	80.44	207	38.86
**PP-PET07**	22.95	155.21	71.43	207	34.51
**PP-PET10**	34.57	154.99	90.18	207	43.56
**PET_PURE**	74.50	252.09	24.05	140	17.18
**PP-PLA00 (@210 °C)**		166.61	98.41	207	47.54
**PP-PLA03**	−3.05	152.14	79.36	207	38.34
**PP-PLA05**	−2.17	153.79	80.90	207	39.08
**PP-PLA07**	−1.95	154.45	81.07	207	39.17
**PP-PLA10**	22.90	151.98	83.82	207	40.49
**PLA_PURE**	88.94	210.34	48.37	93	52.01
**PP-PS00 (@240 °C)**		165.05	94.44	207	45.62
**PP-PS03**	0	152.79	81.45	207	39.35
**PP-PS05**	−1.46	152.62	80.14	207	38.72
**PP-PS07**	0.82	151.75	77.23	207	37.31
**PP-PS10**	−3.91	152.27	78.10	207	37.73
**PS_PURE**	90.68				

## Data Availability

Data are contained within the article.
